# Variable course of disease of rheumatoid arthritis-associated usual interstitial pneumonia compared to other subtypes

**DOI:** 10.1186/s12890-016-0269-2

**Published:** 2016-07-27

**Authors:** Hanna M. Nurmi, Minna K. Purokivi, Miia S. Kärkkäinen, Hannu-Pekka Kettunen, Tuomas A. Selander, Riitta L. Kaarteenaho

**Affiliations:** 1 Center of Medicine and Clinical Research, Division of Respiratory Medicine, Kuopio University Hospital, POB 100, 70029 KYS Kuopio, Finland; 2 Division of Respiratory Medicine, Institute of Clinical Medicine, School of Medicine, Faculty of Health Sciences, University of Eastern Finland, POB 1627, 70211 Kuopio, Finland; 3 Respiratory Medicine, Internal Medicine Research Unit, Medical Research Center Oulu, Oulu University Hospital and University of Oulu, POB 20, 90029 Oulu, Finland; 4 Harjula Hospital, the Municipal Hospital of Kuopio, Niuvantie 4, 70101 Kuopio, Finland; 5 Diagnostic Imaging Center, Division of Radiology, Kuopio University Hospital, POB 100, 70029 Kuopio, Finland; 6 Science Services Center, Kuopio University Hospital, POB 100, 70029 Kuopio, Finland

**Keywords:** High-resolution computed tomography, Cause of death, Comorbidity

## Abstract

**Background:**

In rheumatoid arthritis-associated interstitial lung disease (RA-ILD), occurring in 10 % of patients with patients with RA, usual interstitial pattern (UIP) has shown to associate with poor prognosis but more detailed data about the course of the disease in different subtypes is limited. Our aim was to compare the disease course of patients with RA-ILD categorized into either UIP or other types of ILDs.

**Methods:**

Clinical and radiological information of 59 patients with RA-ILD were re-assessed and re-classified into UIP or non-UIP groups, followed by a between-group comparison of demographic data, lung function, survival, cause of death and comorbidities.

**Results:**

The majority of patients (*n* = 35/59.3 %) showed a radiological UIP-like pattern in high resolution computed tomography. The median survival was 92 months (95 % CI 62.8–121.2) in the UIP-group and 137 months (95 % CI 31.0–243.0) in the non-UIP-group (*p* = 0.417). Differences in course of disease were found in the number of hospitalizations for respiratory reasons (mean 1.9 ± 2.6 in UIP vs. 0.5 ± 0.9 in non-UIP group, *p* = 0.004), the use of oxygen therapy (8/22.9 % UIP patients vs. 0 non-UIP patients, *p* = 0.016), number of deaths (23/65.7 % vs. 10/41.7 %, *p* = 0.046) and decline in diffusion capacity (56 ± 20.6 vs. 69 ± 20.2, *p* = 0.021). Dyspnea and inspiratory crackles were detected more often in the UIP group. RA-ILD was the most common primary cause of death (39.4 % of cases). Hypertension, coronary artery disease, chronic obstructive pulmonary disease, heart insufficiency, diabetes and asthma were common comorbidities. ILD preceded RA diagnosis in 13.6 % of patients.

**Conclusions:**

The course of the disease in RA-UIP patients is different from the other RA-ILD subtypes. Several comorbidities associated commonly with RA-ILD, although ILD was the predominant primary cause of death.

## Background

Interstitial lung disease (ILD) is a rather common extra-articular manifestation of rheumatoid arthritis (RA) and a major cause of morbidity and mortality in RA patients [1, 2]. Approximately 10 % of patients with RA may develop clinically evident ILD with respiratory symptoms and/or a decline in pulmonary function tests [3]. In asymptomatic RA patients, high-resolution computed tomography (HRCT) scans commonly reveal evidence of interstitial lung involvement, and a large proportion of those with subclinical disease deteriorate with time [4, 5]. However, the clinical course of RA-ILD is highly heterogenic, as some patients remain stable for years, even decades, while others develop an insidious progressive disease [6].

While the overall mortality in RA has declined, the numbers of deaths due to RA-ILD have increased [7], although the results of studies investigating survival have been variable. Some studies have reported survival of 3 years, similar to that of idiopathic pulmonary fibrosis (IPF) [8, 9], whereas in others the prognosis of RA-ILD has been significantly better, with median survival of approximately 6–8 years [10, 11].

Since it lacks its own distinctive classification, the subtypes of RA-ILD have been categorized according to the subdivisions of the idiopathic interstitial pneumonias (IIP) [12]. Unlike the situation in other connective tissue diseases (CTD), the most common radiologic and histopathologic pattern of RA-ILD is usual interstitial pneumonia (UIP), whereas nonspecific interstitial pneumonia (NSIP) and other subtypes also exist to a lesser extent [13]. The clinical significance of these different histological and radiological patterns has become nowadays more important since the RA-ILD patient with the UIP pattern (RA-UIP) seems to have a significantly worse prognosis and reduced survival compared to other types such as NSIP and organizing pneumonia (OP) [11, 14–16]. Other differences in the course of the disease in distinct RA-ILD subtypes, in addition to the difference in survival, have not been widely studied so far.

Recently the significance of radiologic and histopathological subtyping of RA-ILD was highlighted as one important area for future investigation [17]. Little is known about concomitant diseases or causes of death of RA-ILD patients. The few studies that have addressed cause of death in these patients, have been unanimous that the majority of deaths are due to respiratory disease either after an exacerbation, infection or simply due to the steady progression of the ILD [11, 13, 18].

The aims of this study were to investigate the numbers and subtypes of the patients with RA-ILD treated in Kuopio University Hospital (KUH), in Eastern Finland, during 2000–2015. The course of the disease, survival, co-morbidities and cause of death were evaluated and compared between UIP and non-UIP cases.

## Methods

### Search and evaluation of data

**Fig. 1:**
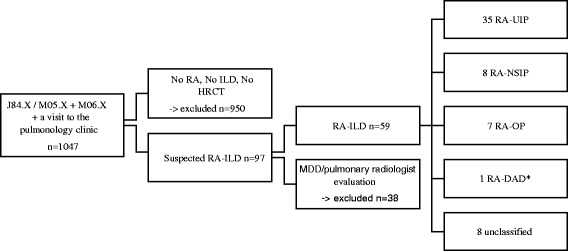
The study protocol and the final categorization of the patients with RA-ILD. *additional 2 DAD findings included in OP group (*n* = 1) and UIP group (*n* = 1)

The subjects for the study were identified from the database of KUH using two International Classification of Diseases (ICD-10) codes, namely J84.X and M05.X/M06.X. (Fig. 1). From these patients we only included the subjects that had been examined or treated in the pulmonology in-patient or out-patient clinic between 1.1.2000 and 31.12.2014 for any respiratory symptoms or any suspected pulmonary disease, thus omitting those RA patients with no symptoms or chest X-ray abnormalities.

A total of 1047 patients were identified and their patient records were evaluated. At baseline, the patients with ILD but without RA (i.e. patients with IIP, other connective tissue disorders (CTD) or allergic alveolitis) and those with RA, whose visits to pulmonology clinic were because of some other lung diseases (such as asthma, chronic obstructive pulmonary disease (COPD), obstructive sleep apnea) were excluded. We also excluded suspected but not confirmed RA-ILD patients, for whom HRCT, or other comparable radiological examination capable of allowing reliable analysis of the lung parenchyma were not available, as were those patients whose RA diagnosis was not certain according to the 1987 classification criteria [19], or who developed later mixed CTD- like symptoms.

Another 38 patients were excluded subsequently after the evaluation by the radiologist and/or after a multidisciplinary discussion due to the very minor signs or nonspecific features for ILD, leaving a total of 59 RA-ILD patients to be studied in detail and classified.

Clinical information was gathered from the patient records of KUH, primary health care centers and other hospitals using a specially designed form. Demographic data included date of birth, sex, occupation, smoking habits, exposure to asbestos, radiation therapy of the thorax region, date of RA diagnosis, date of the first visit to pulmonology clinic due to ILD, comorbidities, death certificates, use of long term oxygen therapy, symptoms and respiratory status findings at baseline, laboratory test results including rheumatoid factor (RF) and antinuclear antibody (ANA) titer and surgery due to RA. Antibodies against cyclic citrullinated peptide were not available for half of the patients. The results of lung function tests, such as spirometry including forced vital capacity (FVC), forced expiratory volume (FEV1) and diffusion capacity to carbon monoxide (DLCO), were gathered at baseline and, when available, during the follow-up at 6 months, 1 year, 2 year and so on annually, including also the most recent available results. Any medication in use prior to ILD diagnosis and also lifelong medication used for RA were recorded. Histological data also was collated. The numbers of hospitalizations due to either respiratory problems (including infections, suspected drug reactions and suspected acute exacerbations) or cardiac problems like unstable angina pectoris, myocardial infarctions, arrhythmias and cardiac failures were collected. Data from death certificates was also collected.

An experienced radiologist evaluated baseline HRCTs from these 59 patients. Radiological ILD categorization was conducted according to the 2013 IIP classification [12]. The radiological RA-UIP criteria were applied from those of IPF [20]. Mainly patients with a definite UIP pattern were included in the UIP group (32 out of 35, 91.4 %). Three patients who displayed a slightly upper (*n* = 2) or mid-lung (*n* = 1) predominated distribution, were included after a multidisciplinary discussion. Patients with possible UIP, i.e. a subpleural and basal predominated reticular abnormality without honeycombing, are not included in the UIP group. When available, an additional HRCT during the follow-up was also evaluated to reveal the progression of the lung disease.

The study protocol was approved by the Ethical Committee of Kuopio University Hospital (statement 17/2013).

### Statistical analysis

The distribution of the continuous variables was verified with Shapiro-Wilk test. If distribution was normally divided, the comparison was made using an independent T-test, otherwise Mann–Whitney U-test was applied. The chi-squared test or Fisher test, when appropriate, was used for categorical variables. Sex, smoking habits, laboratory results and the numbers of deaths are calculated as percentages. Age at the time of RA-ILD diagnosis and lung function results are expressed as mean ± SD. The mean values of the first and most recent available FVCs and DLCOs were calculated in both UIP- and non-UIP groups to determine whether there had been any change in lung function. The mean values of both groups were compared using the independent T-test to evaluate possible differences in lung function tests at the time of RA-ILD diagnosis and also the difference in lung function development. In survival analyses, we excluded the patient who did not have an underlying ILD preceding acute DAD changes. Survival analysis was done using the Kaplan-Meier method and survival curves were compared using the log-rank test. Survival time was calculated from the first visit to the pulmonology clinic due to ILD to the date of death or November 4, 2015 when the vital status was ascertained. Survival results are expressed as median (95 % confidence interval).

We considered a *p*-value <0.05 as statistically significant. All data was analyzed using IBM Statistics SPSS software, version 21.0.

## Results

### Radiologic findings and demographics

Thirty-three (59.5 %) of the patients were male. Most of the patients (*n* = 35/60.3 %) were current or former smokers (Table 1). Five (15.6 %) male and 18 (69.2 %) female patients were never-smokers (*p* < 0.001). The mean age at diagnosis was 66 ± 11.1 years (range 32–87) differing non-significantly in subgroups (UIP vs. non-UIP, non-smokers vs. ever-smokers, male vs. female). RF was positive in 84.2 %, ANA in 17.8 % and antibodies against cyclic citrullinated peptide (CCP) in 60.8 % of the patients.

**Table 1:** Clinical characteristics of the patients with rheumatoid arthritis-associated interstitial lung disease (RA-ILD), which have been classified according to the presence or absence of usual interstitial pneumonia (UIP) pattern in high resolution computed tomography (HRCT)

Characteristics	RA-ILD	RA-UIP	RA-non-UIP	*P*-value
	(*n* = 59)	(*n* = 35, 59.3 %)	(*n* = 24, 40.7 %)	(UIP vs. non-UIP)
Gender				
ᅟMale	33 (55.9)	19 (54.3)	14 (58.3)	0.758
ᅟFemale	26 (44.1)	16 (45.7)	10 (41.7)	
Smoking^a^				
ᅟNever	23 (39.7)	14 (41.2)	9 (37.5)	0.778
ᅟEx-smoker	26 (44.8)	14 (41.2)	12 (50.0)	
ᅟCurrent smoker	9 (15.5)	6 (17.6)	3 (12.5)	
Age (y)	66 ± 11.1	66 ± 11.9	67 ± 10.0	0.597
Serology				
ᅟPositive RF^b^	48 (84.2)	29 (85.3)	19 (82.6)	1.000
ᅟPos. ANA^c^	8 (17.8)	5 (20.0)	3 (15.0)	0.716
ᅟPos. anti-CCP antibody^d^	17 (60.8)	10 (71.4)	7 (63.6)	0.504
Dyspnea^b^	35 (61.4)	25 (73.5)	10 (43.5)	0.022
Cough^e^	31 (60.8)	17 (60.7)	14 (60.9)	0.991
Inspiratory crackles	41 (69.5)	29 (82.9)	12 (50.0)	0.007
FVC % pred	85 ± 17.0	82 ± 17.1	89 ± 16.5	0.164
DLCO % pred	71 ± 18.1	72 ± 20.7	70 ± 13.3	0.635
Medications				
ᅟSteroids, ever	54 (91.5)	32 (91.4)	22 (91.7)	1.000
ᅟMTX, ever	35 (59.3)	18 (51.4)	17 (70.8)	0.136
ᅟMTX, when ILD diagnosed	15 (25.4)	6 (17.1)	9 (37.5)	0.078
ᅟBiological drugs, ever	14 (23.7)	7 (20.0)	7 (29.2)	0.416

Data presented as *n* (percentage) or mean ± SD. *P*-values calculated using Fisher test, *χ* ^2^- test or independent T-test

*RA*-*UIP* usual interstitial pneumonia (UIP) pattern in patients with rheumatoid arthritis (RA). *RA*-*non*-*UIP* Rheumatoid arthritis patients with other than UIP-pattern interstitial lung disease (ILD). *FVC* forced vital capacity. *DLCO* diffusing capacity of the lung for carbon monoxide. *% pred*: percentage of the predicted value. *RF*: rheumatoid factor. *ANA*: anti-nuclear antibodies. *MTX*: methotrexate. *CCP*: cyclic citrullinated peptide

^a^data missing from 1 RA-UIP patient

^b^data missing from 2 patients (1 RA-UIP, 1 RA-non-UIP)

^c^data missing from 14 patients (10 RA-UIP, 4 RA-non-UIP)

^e^data missing from 34 patients (21 RA-UIP, 13 RA-non-UIP)

^f^data missing from 8 patients (7 RA-UIP, 1 RA-non-UIP)

The majority (35/59.3 %) of the patients showed a radiological UIP-pattern in HRCT and the remainder were NSIP (8/13.6 %), OP (7/11.9 %) and 8 patients whose radiological features remained nonspecific, which we termed as unclassified (13.6 %). A diffuse alveolar damage (DAD) pattern was detected in one patient without an underlying ILD, thus likely representing RA-DAD. Additional two DAD patterns were seen in patients with OP and UIP diagnoses prior to DAD.

No statistically significant differences were observed between groups with respect to age, smoking, baseline lung functions or RA serology. Thirty-five (61.4 %) patients suffered from dyspnea and 31 (60.8 %) from cough. Cough was equally common in both groups, but dyspnea occurred more often in the UIP group (*p* = 0.022). Inspiratory crackles were more common in UIP than in non-UIP patients (*p* = 0.007) (Table 1).

### Medication for RA and RA-ILD

Seventy-five percent of patients were receiving some medication for RA at the time of RA-ILD diagnosis (Tables 1 and 2). In 11 cases the RA medication had been markedly changed due to ILD diagnosis. In most cases (9 out of 11), the change was a discontinuation of methotrexate after the diagnosis of ILD. In two patients, either leflunomide or sulfasalazine was discontinued. In all 11 cases (9 UIP, 2 NSIP), ILD continued to progress despite the changes to their RA medication. There were no differences between RA-UIP and RA-non-UIP groups in their use of methotrexate or biological drugs (Table 1). Almost all patients (*n* = 54/91.5 %) had received glucocorticoids at some point.

**Table 2:** The medications of the patients with RA-ILD

Medicine	Ever used for RA or ILD *N* (%)	Used at the time of ILD diagnosis *N* (%)	Discontinued due to ILD diagnosis *N* (%)
Prednisolone	54 (91.5)	10 (16.9)	0 (0.0)
Azathioprine	42 (71.2)	8 (13.6)	0 (0.0)
Methotrexate	35 (59.3)	15 (25.4)	9 out of 15 (60.0)
Hydroxychloroquine	47 (79.7)	16 (27.1)	0 (0.0)
Sulfasalazine	45 (76.3)	16 (27.1)	2 out of 16 (12.5)
Leflunomide	12 (20.3)	2 (3.4)	2 out of 2 (100.0)
Penicillamine	5 (8.5)		
Mycophenolate Mofetil	7 (11.9)		
Sodium aurothiomalate	32 (54.2)	7 (11.9)	0 (0.0)
Cyclosporin	15 (25.4)		
Cyclophosphamide	13 (22.0)		
Chlorambucil	5 (8.5)		
Podophyllotoxin	24 (40.7)	7 (11.9)	0 (0.0)
Etanercept	6 (10.2)		
Infliximab	2 (3.4)		
Golimumab	0 (0.0)		
Adalimumab	5 (8.5)		
Abatacept	1 (1.7)		
Rituximab	11 (18.6)	1 (1.7)	0 (0.0)
Tocilizumab	1 (1.7)		

The first column shows the number of patients receiving each medication at any point and of any duration during their lives. The second column shows the number of patients receiving any particular medication at the time of the RA-ILD diagnosis

Most i.e. 6/7 (85.7 %) RA-OP patients received glucocorticoid treatment for their lung disease and the seventh patient recovered without extra treatment. Of the six steroid-treated RA-OP patients, 5 recovered completely but one did not exhibit a clear beneficial response to treatment. Five of the eight (62.5 %) RA-NSIP patients were treated with high doses of prednisolone two of them enjoying at least a partial response. Two NSIP patients received cyclophosphamide treatment, but both deteriorated despite the treatment. In five RA-UIP patients, high-dose cyclophosphamide plus high-dose steroid treatment was provided but without any positive responses.

### Survival

**Fig. 2:**
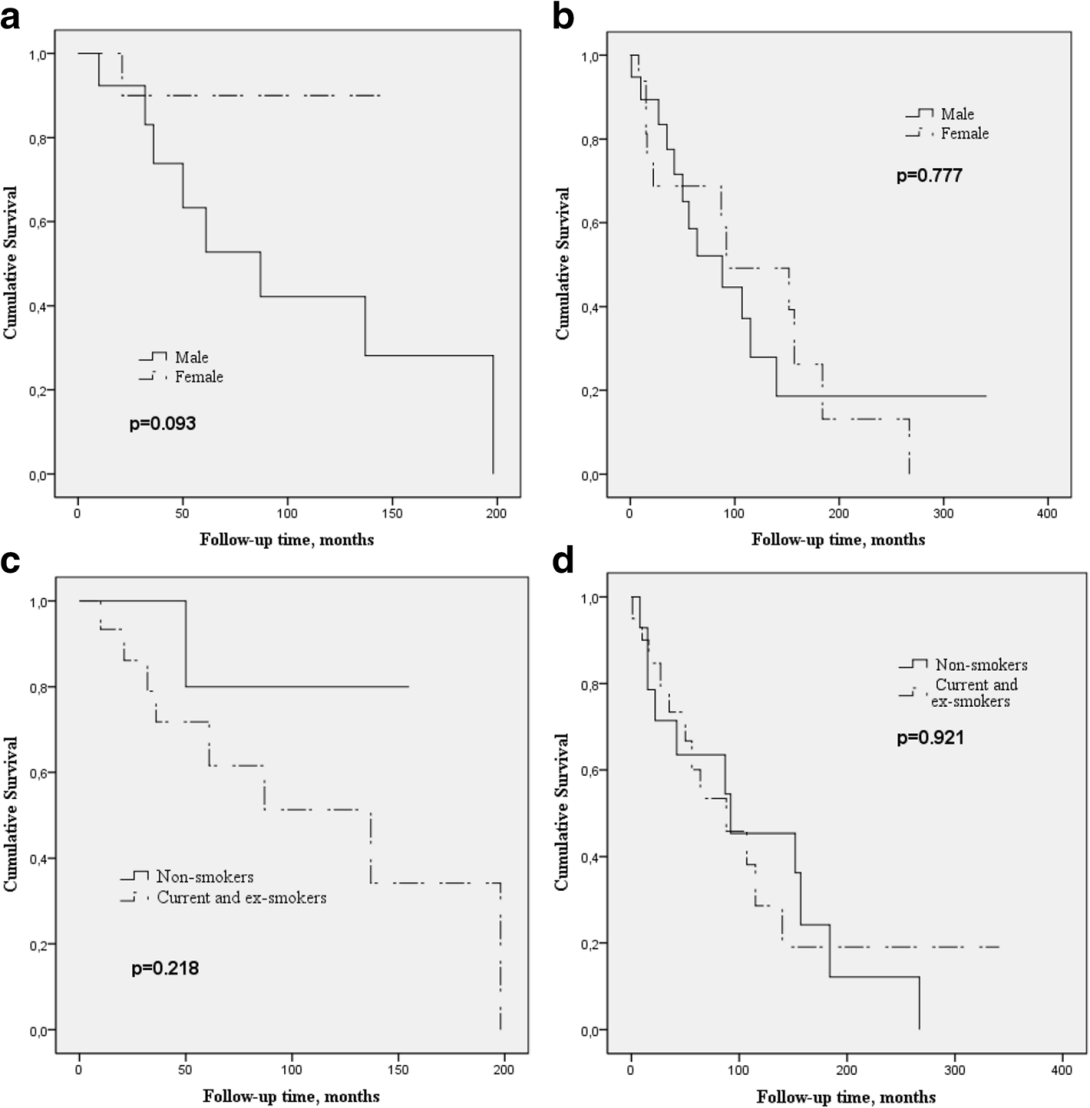
**a**-**d**. Shorter survival (Kaplan-Meier, log-rank) of men was observed in the non-UIP group, but the difference was not quite statistically significant (*p* = 0.093). Survival differences between genders in UIP group were not found (*p* = 0.777). In the non-UIP group, the non-smoking patients seemed to survive for longer than ever-smokers i.e. current smokers and ex-smokers, but the difference did not reach statistical significance (*p* = 0.218). In the UIP group, no differences were found in survival between non-smokers and ever-smokers (*p* = 0.921)

Thirty-three (55.9 %) patients died with median survival of 92.0 months in the UIP and 137.0 months in the non-UIP groups (*p* = 0.417, Table 3). Of the deceased patients, the one with RA-DAD was excluded from the survival analysis. The number of deceased patients was significantly higher in the UIP group, i.e. 23/35 patients with UIP (65.7 %) had died compared with 9/24 (37.5 %) patients with non-UIP (*p* = 0.046, Table 4). Although the median survival in the whole group was longer in women (152 months) than in men (87 months) this difference was not statistically significant (*p* = 0.305). Survival between non-smokers and current/former smokers was also similar in the whole ILD group (*p* = 0.525). Female and non-smoker individuals had a tendency towards longer survival than men and smokers in the non-UIP group, but not in the UIP-group (Table 3, Fig. 2a-d).

**Table 3:** Survival of the patients (months) according to gender and smoking in subgroups

	RA-ILD (*n* = 59)	RA-UIP (*n* = 35, 59.3 %)	RA-non-UIP (*n* = 24, 40.7 %)	*P*-value (UIP vs. non-UIP)
Overall	107.0 (73.1–140.9)	92.0 (62.8–121.2)	137.0 (31.0–243.0)	0.417
Gender				
Male	87.0 (46.0–128.0)	88.0 (31.0–145.0)	87.0 (33.0–141.0)	0.976
Female	152.0 (87.7–216.3)	92.0 (0.0–185.2)	^a^	0.123
	(*p* = 0.305)	(*p* = 0.777)	(*p* = 0.093)	
Smoking				
Non-smokers	152.0 (94.3–209.7)	92.0 (0.0–205.4)	^a^	0.174
Ever-smokers	88.0 (29.4–146.6)	88.0 (30.0–146.0)	137.0 (43.6–230.4)	0.754
	(*p* = 0.525)	(*p* = 0.921)	(*p* = 0.218)	

Data are presented as median (95 % CI). The RA-DAD patient is excluded from the survival analyses

^a^Median survival cannot be calculated since only one death has occurred in this group

**Table 4:** Factors associating with the differential course of disease in the patients with rheumatoid arthritis associated usual interstitial pattern (RA-UIP) and non-UIP patterns (RA-non-UIP)

Factor	RA-ILD	RA-UIP	RA-non-UIP	*P*-value
	(*n* = 59)	(*n* = 35, 59.3 %)	(*n* = 24, 40.7 %)	
Oxygen therapy	8 (13.6)	8 (22.9)	0 (0)	0.016
Hospitalization due to respiratory illness	1.29 ± 2.2 (0–11)	1.9 ± 2.6 (0–11)	0.5 ± 0.9 (0–4)	0.004
Hospitalization due to cardiac illness	0.6 ± 1.2 (0–5)	0.7 ± 1.3 (0–5)	0.4 ± 1.2 (0–4)	0.100
Latest FVC % pred	82 ± 21.2	78 ± 22.9	87 ± 17.2	0.091
(Baseline FVC %)	85 ± 17.0	82 ± 17.1	89 ± 16.5	
Latest DLCO % pred	61 ± 21.3	56 ± 20.6	69 ± 20.2	0.021
(Baseline DLCO %)	71 ± 18.1	72 ± 20.7	70 ± 13.3	
Number of deaths	33 (55.9)	23 (65.7)	9 (37.5)^a^	0.046

Data are presented as percentage or mean ± SD and also (range) in hospitalization

Hospitalization comparison performed using Mann–Whitney U-test and the lung function comparison using an independent sample T-test

^a^One RA-DAD-patient is excluded from this group, *P*-value calculation and survival analyses

### Causes of death

The average age at death was 75.0 ± 9.1 years, ranging from 54.8 to 91.7 years. The UIP patients died slightly younger than their non-UIP counterparts (73.6 ± 9.8 vs. 78.2 ± 6.5, *p* = 0.187).

**Fig. 3:**
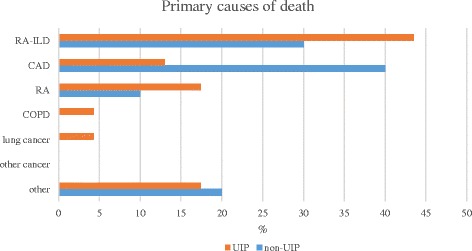
ILD was the major cause of death in the UIP group (10/43.5 %), whereas that of the non-UIP group was cardiovascular disease (4/40.0 %). None of the differences reached statistical significance. CAD : coronary artery disease, COPD: chronic obstructive pulmonary disease, RA: rheumatoid arthritis

According to the death certificates of the 33 deceased patients, RA-ILD was the most common primary cause of death; 13/39.4 % cases (10 UIP, 3 non-UIP; *p* = 0.701), (Fig. 3). RA-ILD was primary cause of death equally in men and women (8 vs. 5, *p* = 1.000) and in non-smokers and ever-smokers (6 vs. 7, *p* = 0.522). Coronary artery disease (CAD) was the second most common primary cause of death in 7 individuals (21.2 %; 3 UIP, 4 non-UIP, *p* = 0.161). RA was the primary cause of death in 5 cases (15.2 %). In the other cases, the primary causes of death were Alzheimer’s disease, universal atherosclerotic disease with acute ischemia in legs, acute pancreatitis, intestinal tuberculosis, chronic obstructive pulmonary disease (COPD), massive bleeding due to pelvic fracture, lung cancer and suspected viral infection in the central nervous system – each one case.

Pneumonia and CAD were equally common as the immediate cause of death (both 10/30.3 %; 6 UIP, 4 non-UIP) and RA-ILD (5/15.2 %; 4 UIP, 1 non-UIP) was also prevalent. Lung cancer, RA, diabetes, RA associated secondary amyloidosis with renal failure, acute pancreatitis, diabetes, intestinal tuberculosis and gastroenteritis represented immediate causes of death of single cases.

### Comorbidities

**Fig. 4:**
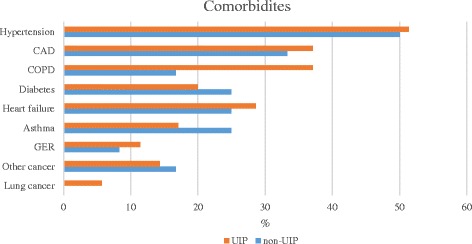
The most common comorbidities were hypertension, coronary artery disease (CAD), COPD, diabetes and heart failure, although asthma was also relatively common. COPD occurred more often in patients with UIP (13/37.1 % UIP vs. 4/16.7 % non-UIP, *p* = 0.088). CAD: coronary artery disease, COPD: chronic obstructive pulmonary disease, GER: gastroesophageal reflux

The most common comorbidities were hypertension (30/50.8 %), CAD (21/35.6 %), COPD (17/28.8 %), heart insufficiency (16/27.1 %), diabetes (13/22.0 %) and asthma (12/20.3 %) (Fig. 4). Gastroesophageal reflux (GER) occurred in 6 (10.2 %) and hypothyroidism in 3 (5.1 %) patients. There were two lung cancers (3.4 %) and 9 (15.3 %) other cancers including basal cell carcinoma (*n* = 2), diffuse large B cell lymphoma (*n* = 2), urinary bladder carcinoma (*n* = 1), colon adenocarcinoma (*n* = 1), squamous cell carcinoma in upper lip (*n* = 1) and in tongue (*n* = 1) and carcinoma in ventricle (*n* = 1). Most of the cancers were not primary causes of death. Six (10.2 %) patients suffered from tuberculosis (five lung and one intestinal). No statistically significant differences in the comorbidities were found between UIP and non-UIP groups, although COPD was more common in the UIP group (*p* = 0.088). Asthma was more common in women (*p* = 0.016) and COPD in men (*p* < 0.001). Comorbidities divided equally between non-smokers and ever-smokers, except for COPD (*p* < 0.001).

### Timing of diagnosis

In eight patients (13.6 %), ILD preceded RA diagnosis. In three of these cases (2 UIP, 1 OP) the RA diagnosis was made within one year after the ILD diagnosis, but in five cases (3 UIP, 1 OP, 1 unclassified) joint symptoms and RA diagnosis appeared over one year after the ILD diagnosis (range 2.17–9.58 years). In two cases (3.4 %) RA and ILD were diagnosed simultaneously. The RA diagnosis date was missing in one case. ILD followed the diagnosis of RA in 48 patients after a variable period of time i.e. 6/12.5 % within a year, 13/27.1 % within 3 years and 19/39.6 % within 5 years. The longest time interval between RA and ILD was 52.1 years.

### Course of disease

Several factors were indicative of ILD progression i.e. oxygen treatment, hospitalizations and decline of diffusion capacity to carbon monoxide (DLCO) (Table 4). All patients (*n* = 8) using oxygen therapy belonged to the UIP group (*p* = 0.016). The number of hospitalizations due to respiratory causes was significantly higher in UIP compared to non-UIP (*p* = 0.004). The latest available DLCO results were significantly lower in UIP (*p* = 0.021). Forced vital capacity (% predicted) (FVC %) showed a trend towards a greater decline in the UIP group, (*p* = 0.091).

## Discussion

This study revealed that the course of disease in the patients with RA-ILD was variable in subtypes categorized according to either the presence or absence of the UIP-pattern in HRCT. The patients with RA-UIP used oxygen, suffered from hospitalizations due to respiratory reasons and suffered an accelerated decline of lung function more often than those with non-UIP subtype. Moreover, several comorbidities were very common, and in addition to RA-ILD, CAD was a common primary cause of death.

The distribution of genders was almost equal supporting previous findings that male sex is a risk factor for ILD [5, 8, 21], minding that RA is twice as common in females [22]. The proportion of patients with UIP (59.3 %) and the amount of cases (13.7 %) in which ILD preceded articular disease were similar as described recently [21]. Dyspnea and inspiratory crackles were more common in the patients with UIP, in agreement with previous results [23]. The lung disease was more progressive in the UIP group based on the number of deaths, use of oxygen, hospitalization due respiratory reasons and decline of pulmonary function, especially DLCO. Some of the hospitalizations may have been attributable to acute exacerbations, known to occur mostly in UIP patterned RA-ILD [24]. In summary, our findings support previous studies suggesting that RA-UIP follows a distinctive pathological course [13, 25].

ILD was the primary cause of death in the majority of subjects, especially in the UIP group, although this did not reach statistical significance in our small study population. A previous study also indicated that RA-ILD patients were most likely to die of ILD or RA itself [7]. A recent Finnish study revealed that CAD was responsible for 43 % of deaths of RA patients [26] whereas in Korea, malignancies were the major cause of death in these patients [27]. The high percentage (39.4 %) of ILD as a primary cause of death indicates that even though several comorbidities often coexist, ILD remains the leading cause of death. The immediate causes of deaths did not exhibit any significant differences between the UIP and non-UIP groups.

CAD was a major comorbidity in RA-ILD. Previously, the risk of CAD and hypertension has been shown to increase in RA already at disease onset [28]. One novel finding was that asthma was more common in females, although an association between asthma and RA has been previously detected [29]. COPD was observed in almost 30 % of patients, in line with a recent study revealing a 48 % prevalence of emphysema [30]. COPD was more common in men, although this may be attributable to different smoking habits between the genders. COPD was also more prevalent in UIP patients even though smoking was similar in both groups. GER, previously claimed to be associated with IPF [31], or hypothyroidism thought to be more common in RA [32], were not prevalent in our study.

Previously published studies of survival of the patients with RA-ILD have revealed variable results. Some have reported survival as being as poor as in IPF i.e. approximately 3 years [8, 33, 34] but others have revealed longer survival times i.e. 7–8 years [10, 11], durations in line with the present study. Furthermore, the lifespan of RA-UIP has been shown to be shorter than that of the other subtypes [14]. The median survival in our study was shorter in patients with UIP than in their non-UIP counterparts (92 vs 137 months) but this result did not reach statistical significance. Male gender has been recognized as a risk factor for RA-ILD mortality in previous studies [35]. In our study, survival analyses revealed a tendency that non-UIP, but not UIP, females and non-smokers, lived longer.

Identifying the RA-ILD patients from hospital registers was challenging since two different diagnosis codes were needed and, moreover, medical records of hundreds of patients had to be reviewed before we could gather this study population, which is similar in size as the majority of published reports, except for a few multicenter studies [21]. In fact, this sample size can be considered as representative since approximately 248,400 people live in the KUH region. In addition, we intentionally excluded the patients with only minor changes in HRCT since our purpose was to study the verifiably clinically relevant RA-ILD. The retrospective protocol of the data collection may have caused some inaccuracies and missing data. Categorization into either UIP or non-UIP groups was based on radiological evaluation, since histological data was limited. The radiological categorization can nonetheless be considered as reasonably reliable, since a definite UIP pattern in a HRCT scan has been demonstrated to be a sensitive and specific way of detecting the histopathologic UIP pattern in both IPF and RA-ILD [36–38]. Therefore we are confident that the UIP group reliably consists of true RA-UIP patients, although it is possible that some of the patients in the NSIP or unclassified group may be suffering from histological UIP. One obvious limitation of this study is the fact that the re-categorization of the patients was performed by one radiologist. However, a large proportion of the HRCT scans were evaluated in a multidisciplinary discussion. In this study, due to its retrospective nature, it was not possible to evaluate thoroughly the effects of therapeutic interventions since the patients had received highly variable treatments for RA and ILD without being followed with a standardized protocol as was also the case in a previously published investigation [39].

## Conclusions

In summary, we detected several differences in disease course between RA-UIP and RA-non-UIP confirming the existing impression, that the UIP patterned ILD is more severe than the other subtypes of RA-ILD. In addition, even though several comorbidities often coexist with RA-ILD, the ILD itself seems to cause the majority of the deaths in these patients.

## Abbreviations

ANA, antinuclear antibodies; CAD, coronary artery disease; CCP, cyclic citrullinated peptide; CI, confidence interval; COPD, chronic obstructive pulmonary disease; CTD, connective tissue diseases; DAD, diffuse alveolar damage; DLCO, diffusion capacity to carbon monoxide; FVC, forced vital capacity; GER, gastro-esophageal reflux; HRCT, high-resolution computed tomography; IIP, idiopathic interstitial pneumonias; ILD, interstitial lung disease; IPF, idiopathic pulmonary fibrosis; KUH, Kuopio University Hospital; MDD, multidisciplinary discussion; NSIP, nonspecific interstitial pneumonia; OP, organizing pneumonia; RA, rheumatoid arthritis; RA-DAD, rheumatoid arthritis-associated diffuse alveolar damage; RA-ILD, rheumatoid arthritis-associated interstitial pneumonia; RA-NSIP, rheumatoid arthritis-associated nonspecific interstitial pneumonia; RA-OP, rheumatoid arthritis-associated organizing pneumonia; RA-UIP, rheumatoid arthritis-associated usual interstitial pneumonia; RF, rheumatoid factor; SD, standard deviation; UIP, usual interstitial pneumonia
